# Crystal structure and Hirshfeld surface analysis of ethyl 2-({5-acetyl-3-cyano-6-methyl-4-[(*E*)-2-phenyl­ethen­yl]pyridin-2-yl}sulfan­yl)acetate

**DOI:** 10.1107/S2056989021005600

**Published:** 2021-06-25

**Authors:** Safiyyah A. H. Al-Waleedy, Shaaban K. Mohamed, Joel T. Mague, Mehmet Akkurt, Mohamed S. Abbady, Etify A. Bakhite

**Affiliations:** a Department of Chemistry, Faculty of Science, Taiz University, Taiz, Yemen; b Chemistry and Environmental Division, Manchester Metropolitan University, Manchester, M1 5GD, England; c Chemistry Department, Faculty of Science, Minia University, 61519 El-Minia, Egypt; d Department of Chemistry, Tulane University, New Orleans, LA 70118, USA; e Department of Physics, Faculty of Sciences, Erciyes University, 38039 Kayseri, Turkey; f Chemistry Department, Faculty of Science, Assiut University, Assiut 71516, Egypt

**Keywords:** crystal structure, pyridine, styr­yl, ester, hydrogen bond, Hirshfeld surface analysis

## Abstract

In the title mol­ecule, C_21_H_20_N_2_O_3_S, the styryl and ester substituents are displaced to opposite sides of the plane of the pyridine ring. In the crystal, C—H⋯O hydrogen bonds form chains extending parallel to the *a-*axis direction, which pack with partial inter­calation of the styryl and ester substituents. A Hirshfeld surface analysis indicates that the most significant contributions to the crystal packing are from H⋯H (43.6%), C⋯H/H⋯C (15.6%), O⋯H/H⋯O (14.9%) and N⋯H/H⋯N (11.2%) contacts.

## Abstract 2

The styryl and ester substituents are displaced to opposite sides of the plane through the pyridine ring while the acetyl group is rotated well out of that plane. In the crystal, inversion-related C—H⋯O hydrogen bonds form chains extending parallel to the *a-*axis direction, which pack with partial inter­calation of the styryl and ester substituents.

## Chemical context   

Numerous pyridine-containing natural products and synthetic organic compounds with various biophysio- and pharmacological activities have been reported (Gibson *et al.*, 2007 ▸; Vidaillac *et al.*, 2007 ▸). These scaffolds are also of widespread inter­est in supra­molecular and coordination chemistry, as well as for materials science (Balasubramanian & Keay, 1996 ▸). The above findings promoted us to study the crystal structure of the title compound, C_21_H_20_N_2_O_3_S.
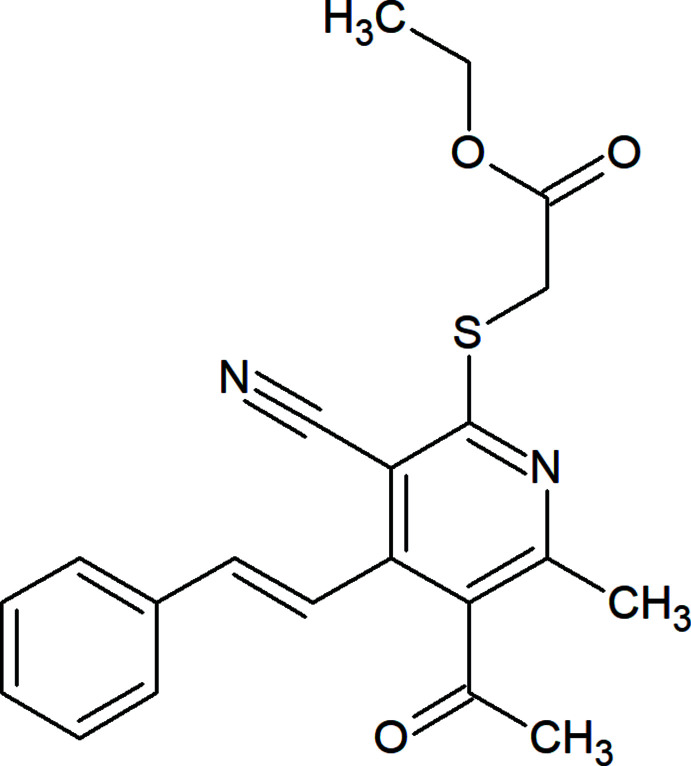


## Structural commentary   

**Figure 1:**
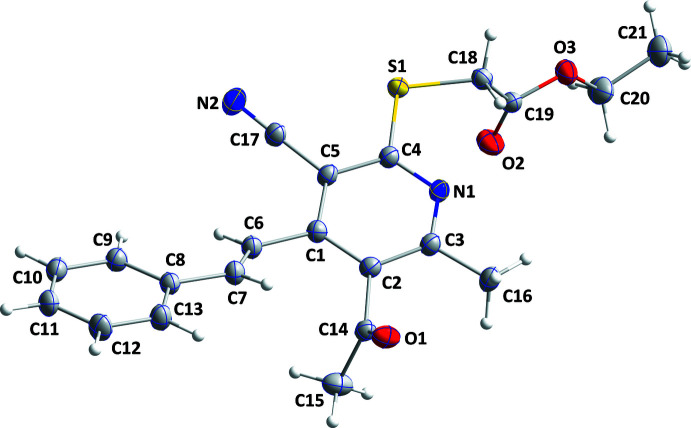
The title mol­ecule with labelling scheme and displacement ellipsoids at the 50% probability level.

The styryl substituent and the ester group are displaced to opposite sides of the plane of the pyridine ring (Fig. 1 ▸). The dihedral angle between the mean planes of the phenyl (C8–C13) and pyridine (N1/C1–C5) rings is 27.86 (3)°. The C1—C2—C14—C15 torsion angle of 68.1 (2)° indicates that the acetyl group is rotated well out of the plane of the pyridine ring, while the N1—C4—S1—C18 torsion angle of −5.66 (12)° shows that the link to the ester group is nearly coplanar with the pyridine ring.

## Supra­molecular features   

**Figure 2:**
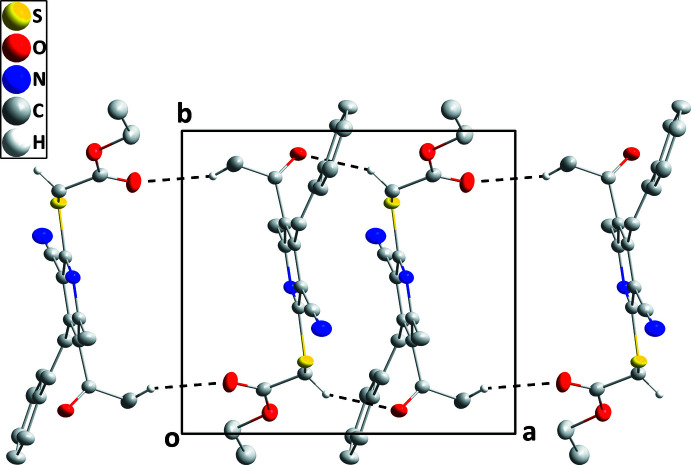
A portion of one hydrogen–bonded chain in a view along the *c-*axis direction. C—H⋯O hydrogen bonds are depicted by dashed lines.

**Figure 3:**
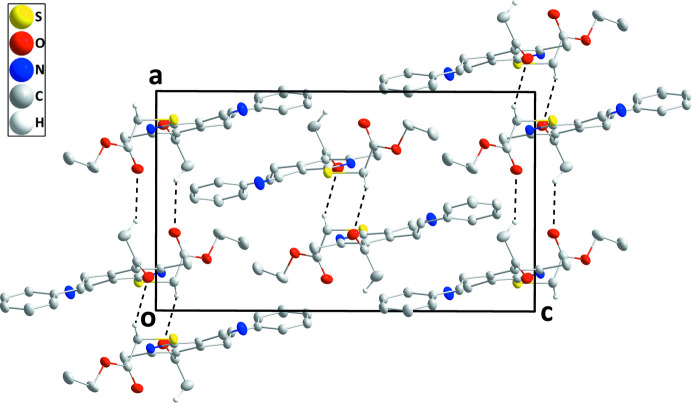
Packing of the mol­ecules in the title compound in a view along the *b*-axis direction. C—H⋯O hydrogen bonds are depicted by dashed lines.

In the crystal, inversion dimers are formed by inter­molecular C15—H15*A*⋯O2 hydrogen bonds between a methyl H atom of the acetyl group and the carbonyl O atom of the ester function. These dimers are further linked by inversion-related C18—H18*B*⋯O1 hydrogen bonds between a methyl­ene H atom and the carbonyl O atom of the acetyl group (Table 1 ▸) to form ribbons of mol­ecules extending parallel to the *a*-axis direction (Fig. 2 ▸). The chains pack with a partial inter­calation of the styryl and ester substituents (Fig. 3 ▸).

## Hirshfeld surface analysis   

**Figure 4:**
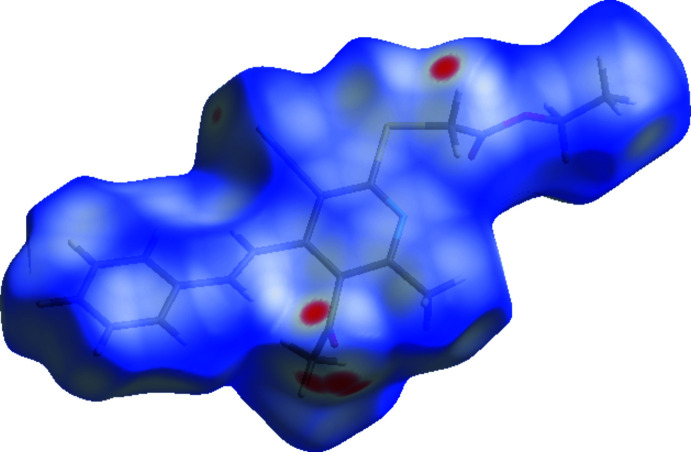
A view of the three-dimensional Hirshfeld surface for the title compound, plotted over *d*_norm_ in the range −0.1607 to +1.3888 a.u.

**Figure 5:**
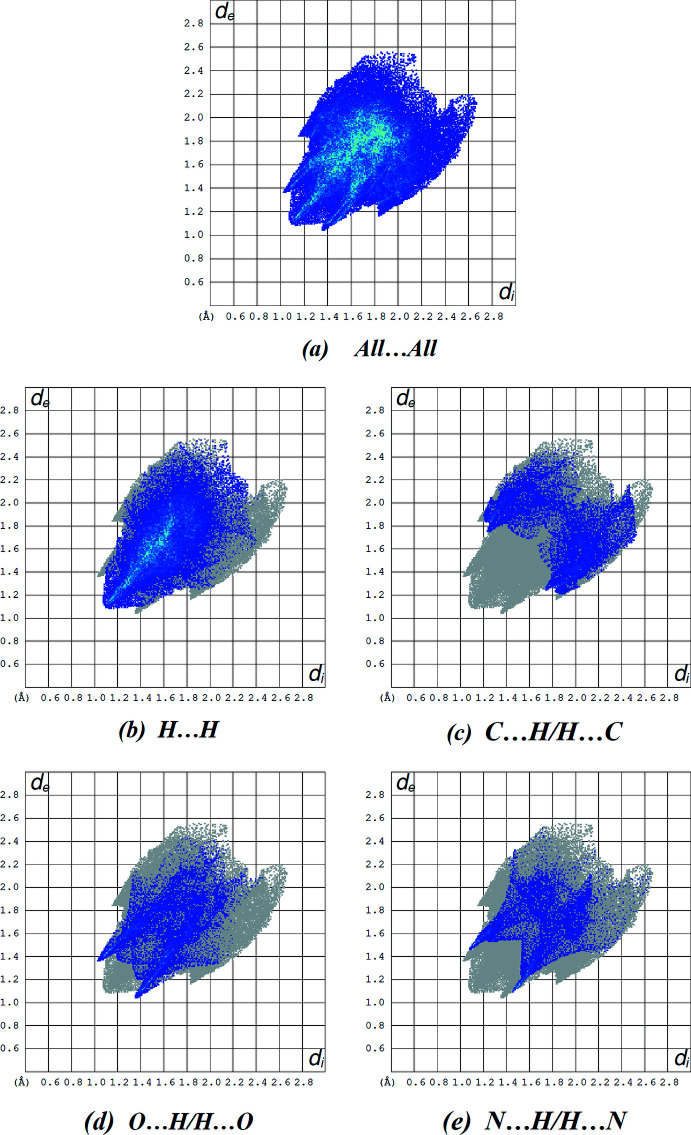
A view of the two-dimensional fingerprint plots for the title compound, showing (*a*) all inter­actions, and delineated into (*b*) H⋯H, (*c*) C⋯H/H⋯C, (*d*) O⋯H/H⋯O and (*e*) N⋯H/H⋯N inter­actions. The *d*_i_ and *d*_e_ values are the closest inter­nal and external distances (in Å) from given points on the Hirshfeld surface.

To qu­antify the inter­molecular inter­actions in the title compound, a Hirshfeld surface analysis was performed and two-dimensional fingerprint plots were generated using *Crystal Explorer* (Turner *et al.*, 2017 ▸). The Hirshfeld surface mapped over *d*_norm_ in the range −0.1607 to +1.3888 arbitrary units is depicted in Fig. 4 ▸, where the red regions indicate apparent hydrogen bonds in this structure. The intensities of the red areas are greater for C15—H15*A*⋯O2 and C18—H18*B*⋯O1, indicating the strongest inter­actions as compared to other red spots on the Hirshfeld surface; Table 2 ▸ lists corresponding close inter­molecular contacts. The two-dimensional fingerprint plots (Fig. 5 ▸) reveal that the largest contributions are from H⋯H (43.6%; Fig. 5 ▸ *b*), C⋯H/H⋯C (15.6%; Fig. 5 ▸ *c*), O⋯H/H⋯O (14.9%; Fig. 5 ▸ *d*) and N⋯H/H⋯N (11.2%; Fig. 5 ▸ *e*) inter­actions. Other inter­actions contributing less to the crystal packing are S⋯H/H⋯S (5.9%), C⋯C (4.4%), N⋯C/C⋯N (1.5%), S⋯O/O⋯S (1.1%), O⋯C/C⋯O (1.0%), O⋯O (0.3%), N⋯N (0.2%) and S⋯C/C⋯S (0.2%).

## Database survey   

A search of the Cambridge Structural Database (version 5.42, update 1, Feb 2021; Groom *et al.*, 2016 ▸) for related structures with the 2-sulfanyl­pyridine-3-carbo­nitrile moiety of the title compound gave the following matches: ethyl 4-methyl-2-phenyl-6-thioxo-1,6-di­hydro-5-pyrimidine­carboxyl­ate monohydrate (DEWCIS; Cunha *et al.*, 2007 ▸), ethyl 4-(5-eth­oxy­carbonyl-6-methyl-2-phenyl-4-pyrimidinyldisulfan­yl)-6-meth­yl-2-phenyl-5-pyrimidine­carboxyl­ate (DEWCAK; Cunha *et al.*, 2007 ▸), ethyl 4-{[(4-chloro­phen­yl)meth­yl]sulfan­yl}-6-meth­yl-2-phenyl­pyrimidine-5-carboxyl­ate (NILKOL; Stolarczyk *et al.*, 2018 ▸), (4-{[(4-chloro­phen­yl)meth­yl]sulfan­yl}-6-methyl-2-phenyl­pyrimidin-5-yl)methanol (NILKUR; Stolarczyk *et al.*, 2018 ▸) and 4-{[(4-chloro­phen­yl)meth­yl]sulfan­yl}-5,6-dimethyl-2-phenyl­pyrimidine (NILLAY; Stolarczyk *et al.*, 2018 ▸).

Compound DEWCIS crystallizes in the space group *P*2_1_/*c* with one mol­ecule in the asymmetric unit. N—H⋯O, O—H⋯N and O—H⋯S inter­actions involving the water mol­ecules, as well as π–π stacking inter­actions between the mol­ecules along the *b* axis contribute to the formation of layers parallel to the *bc* plane. The stability of the mol­ecular packing is achieved by van der Waals inter­actions between these layers. Compound DEWCAK crystallizes in the space group *P* 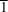 with one mol­ecule in the asymmetric unit. In the crystal structure of DEWCAK, there are no classical hydrogen bonds. The mol­ecular packing is stabilized by C—H⋯π inter­actions and π–π stacking inter­actions. Compound NILKOL crystallizes in the space group *P* 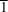 with one mol­ecule in the asymmetric unit, whereas compounds NILKUR and NILLAY crystallize in the space group *P*2_1_/*c* with two and one mol­ecules, respectively, in their asymmetric units. The conformation of each mol­ecule is best defined by the dihedral angles formed between the pyrimidine ring and the planes of the two aryl substituents attached at the 2- and 4-positions. The only structural difference between the three compounds is the substituent at the 5-position of the pyrimidine ring, but they present significantly different features in their hydrogen-bonding inter­actions. NILKOL displays a chain structure whereby the chains are further extended into a two-dimensional network. In NILKUR and NILLAY, the hydrogen-bonded chains have no further aggregation.

## Synthesis and crystallization   

A mixture of 5-acetyl-3-cyano-6-methyl-4-styryl­pyridine-2(1*H*)-thione (3.24 g, 10 mmol), ethyl chloro­acetate (1.1 ml, 10 mmol) and sodium acetate trihydrate (1.5 g, 11 mmol) in ethanol (40 ml) was heated under reflux for 30 min. The solid that formed after dilution with water (20 ml) was filtered off and recrystallized from methanol in the form of fine colourless crystals of the title compound, yield 85%; m.p. 343–344 K. Its IR spectrum showed characteristic absorption bands at 2219 cm^−1^ for (C≡N), at 1748 cm^−1^ for (C=O, non conjugated ester) and at 1724 cm^−1^ for (C=O, conjugated ester). The ^1^H NMR spectrum (400 MHz, DMSO-*d*_6_) displayed a multiplet at δ = 6.60–7.63 ppm (7H: CH=CH and Ar-Hs), a multiplet at δ = 4.16–4.37 ppm (6H: two OCH_2_ and SCH_2_), a singlet at δ = 2.52 ppm (3H, CH_3_ at C-6, overlapped with solvent signal) and a multiplet at δ = 1.21–1.27 ppm (6H: two CH_3_ of ester groups).

## Refinement   

Crystal data, data collection and structure refinement details are summarized in Table 3 ▸. The C-bound H atoms were refined freely, while the H atoms of the C16 methyl group were placed geometrically (C—H = 0.98 Å) and refined as riding atoms with *U*_iso_(H) = 1.5*U*_eq_(C).

## Supplementary Material

Crystal structure: contains datablock(s) I, global. DOI: 10.1107/S2056989021005600/wm5610sup1.cif

Structure factors: contains datablock(s) I. DOI: 10.1107/S2056989021005600/wm5610Isup2.hkl

Supporting information file. DOI: 10.1107/S2056989021005600/wm5610Isup3.cml

CCDC reference: 2087180

Additional supporting information:  crystallographic information; 3D view; checkCIF report

## Figures and Tables

**Table 1:** Hydrogen-bond geometry (Å, °)

*D*—H⋯*A*	*D*—H	H⋯*A*	*D*⋯*A*	*D*—H⋯*A*
C15—H15*A*⋯O2^i^	0.98 (2)	2.56 (2)	3.375 (2)	139.9 (17)
C18—H18*B*⋯O1^ii^	0.965 (17)	2.493 (17)	3.2989 (17)	140.9 (13)

Symmetry codes: (i) -x+2, -y+1, -z+1; (ii) -x+1, -y+1, -z+1.

**Table 2:** Summary of short inter­atomic contacts (Å) in the title compound

Contact	Distance	Symmetry operation
H20*B*⋯H16*B*	2.53	*x*, 1 + *y*, *z*
H18*B*⋯H7	2.42	1 − *x*, 1 − *y*, 1 − *z*
O2⋯H10	2.613	{3\over 2} − *x*, {1\over 2} + *y*, {1\over 2} − *z*
H15*A*⋯O2	2.56	2 − *x*, 1 − *y*, 1 − *z*
N2⋯H20*A*	2.63	−{1\over 2} + *x*, {3\over 2} − *y*, −{1\over 2} + *z*
H11⋯H11	2.31	1 − *x*, −*y*, −*z*
H12⋯H20*A*	2.47	−{1\over 2} + *x*, {1\over 2} − *y*, −{1\over 2} + *z*
H16*A*⋯H21*B*	2.49	{3\over 2} − *x*, −{1\over 2} + *y*, {3\over 2} − *z*

**Table 3:** Experimental details

Crystal data	
Chemical formula	C_21_H_20_N_2_O_3_S
*M*_r_	380.45
Crystal system, space group	Monoclinic, *P*2_1_/*n*
Temperature (K)	150
*a*, *b*, *c* (Å)	10.7365 (4), 9.7590 (3), 18.5600 (7)
β (°)	90.066 (1)
*V* (Å^3^)	1944.67 (12)
*Z*	4
Radiation type	Cu *K*α
μ (mm^−1^)	1.67
Crystal size (mm)	0.27 × 0.12 × 0.05

Data collection	
Diffractometer	Bruker D8 VENTURE PHOTON 100 CMOS
Absorption correction	Multi-scan (*SADABS*; Krause *et al.*, 2015 ▸)
*T*_min_, *T*_max_	0.80, 0.92
No. of measured, independent and observed [*I* > 2σ(*I*)] reflections	14722, 3912, 3520
*R*_int_	0.034
(sin θ/λ)_max_ (Å^−1^)	0.625

Refinement	
*R*[*F*^2^ > 2σ(*F* ^2^)], *wR*(*F* ^2^), *S*	0.033, 0.088, 1.04
No. of reflections	3912
No. of parameters	314
H-atom treatment	H atoms treated by a mixture of independent and constrained refinement
Δρ_max_, Δρ_min_ (e Å^−3^)	0.22, −0.29

Computer programs: *APEX3* and *SAINT* (Bruker, 2016 ▸), *SHELXT* (Sheldrick, 2015*a* ▸), *SHELXL* (Sheldrick, 2015*b* ▸), *DIAMOND* (Brandenburg & Putz, 2012 ▸) and *publCIF* (Westrip, 2010 ▸).

## supplementary crystallographic information

### Crystal data

**Table:**

C_21_H_20_N_2_O_3_S	*F*(000) = 800
*M**_r_* = 380.45	*D*_x_ = 1.299 Mg m^−^^3^
Monoclinic, *P*2_1_/*n*	Cu *K*α radiation, λ = 1.54178 Å
*a* = 10.7365 (4) Å	Cell parameters from 9951 reflections
*b* = 9.7590 (3) Å	θ = 4.8–74.6°
*c* = 18.5600 (7) Å	µ = 1.67 mm^−^^1^
β = 90.066 (1)°	*T* = 150 K
*V* = 1944.67 (12) Å^3^	Column, colourless
*Z* = 4	0.27 × 0.12 × 0.05 mm

### Data collection

**Table:**

Bruker D8 VENTURE PHOTON 100 CMOS diffractometer	3912 independent reflections
Radiation source: INCOATEC IµS micro–focus source	3520 reflections with *I* > 2σ(*I*)
Mirror monochromator	*R*_int_ = 0.034
Detector resolution: 10.4167 pixels mm^-1^	θ_max_ = 74.6°, θ_min_ = 4.8°
ω scans	*h* = −13→12
Absorption correction: multi-scan (*SADABS*; Krause *et al.*, 2015)	*k* = −11→12
*T*_min_ = 0.80, *T*_max_ = 0.92	*l* = −21→22
14722 measured reflections	

### Refinement

**Table:**

Refinement on *F*^2^	Secondary atom site location: difference Fourier map
Least-squares matrix: full	Hydrogen site location: mixed
*R*[*F*^2^ > 2σ(*F*^2^)] = 0.033	H atoms treated by a mixture of independent and constrained refinement
*wR*(*F*^2^) = 0.088	*w* = 1/[σ^2^(*F*_o_^2^) + (0.0426*P*)^2^ + 0.7692*P*] where *P* = (*F*_o_^2^ + 2*F*_c_^2^)/3
*S* = 1.04	(Δ/σ)_max_ = 0.001
3912 reflections	Δρ_max_ = 0.22 e Å^−^^3^
314 parameters	Δρ_min_ = −0.28 e Å^−^^3^
0 restraints	Extinction correction: *SHELXL 2018/3* (Sheldrick, 2015*b*), Fc^*^=kFc[1+0.001xFc^2^λ^3^/sin(2θ)]^-1/4^
Primary atom site location: dual	Extinction coefficient: 0.0041 (3)

### Special details

**Table:**

Geometry. All esds (except the esd in the dihedral angle between two l.s. planes) are estimated using the full covariance matrix. The cell esds are taken into account individually in the estimation of esds in distances, angles and torsion angles; correlations between esds in cell parameters are only used when they are defined by crystal symmetry. An approximate (isotropic) treatment of cell esds is used for estimating esds involving l.s. planes.
Refinement. Refinement of F^2^ against ALL reflections. The weighted R-factor wR and goodness of fit S are based on F^2^, conventional R-factors R are based on F, with F set to zero for negative F^2^. The threshold expression of F^2^ > 2sigma(F^2^) is used only for calculating R-factors(gt) etc. and is not relevant to the choice of reflections for refinement. R-factors based on F^2^ are statistically about twice as large as those based on F, and R- factors based on ALL data will be even larger. Independent refinement of the hydrogen atoms attached to C16 led to an unreasonable geometry so these were included as riding contributions (C—H = 0.98 Å) with an AFIX 137 instruction.

### Fractional atomic coordinates and isotropic or equivalent isotropic displacement parameters (Å^2^)

**Table:**

	*x*	*y*	*z*	*U*_iso_*/*U*_eq_	
S1	0.62964 (3)	0.76240 (3)	0.45327 (2)	0.02319 (11)	
O1	0.65133 (9)	0.07606 (9)	0.47995 (6)	0.0295 (2)	
O2	0.85513 (10)	0.82982 (12)	0.54910 (6)	0.0367 (3)	
O3	0.73641 (9)	0.93032 (11)	0.63373 (5)	0.0304 (2)	
N1	0.67271 (10)	0.51703 (11)	0.51422 (6)	0.0222 (2)	
N2	0.58138 (13)	0.65298 (13)	0.27261 (7)	0.0351 (3)	
C1	0.66330 (11)	0.37425 (13)	0.38230 (7)	0.0203 (3)	
C2	0.69019 (12)	0.30604 (13)	0.44698 (7)	0.0202 (3)	
C3	0.69103 (12)	0.38044 (13)	0.51167 (7)	0.0215 (3)	
C4	0.65042 (12)	0.58371 (13)	0.45305 (7)	0.0201 (3)	
C5	0.64281 (12)	0.51610 (13)	0.38647 (7)	0.0208 (3)	
C6	0.64972 (13)	0.30725 (14)	0.31165 (7)	0.0233 (3)	
H6	0.6751 (17)	0.363 (2)	0.2711 (10)	0.038 (5)*	
C7	0.59846 (13)	0.18469 (14)	0.30086 (7)	0.0239 (3)	
H7	0.5717 (16)	0.1327 (18)	0.3420 (10)	0.033 (4)*	
C8	0.57238 (12)	0.12114 (13)	0.23083 (7)	0.0222 (3)	
C9	0.58480 (13)	0.19075 (14)	0.16518 (7)	0.0245 (3)	
H9	0.6136 (17)	0.284 (2)	0.1648 (9)	0.034 (5)*	
C10	0.55575 (13)	0.12655 (16)	0.10077 (8)	0.0276 (3)	
H10	0.5629 (17)	0.178 (2)	0.0567 (10)	0.041 (5)*	
C11	0.51380 (14)	−0.00811 (16)	0.10078 (8)	0.0295 (3)	
H11	0.4913 (18)	−0.0529 (19)	0.0555 (10)	0.040 (5)*	
C12	0.50215 (15)	−0.07840 (15)	0.16501 (8)	0.0312 (3)	
H12	0.4716 (17)	−0.170 (2)	0.1652 (10)	0.037 (5)*	
C13	0.53114 (14)	−0.01448 (15)	0.22967 (8)	0.0279 (3)	
H13	0.5240 (17)	−0.0659 (18)	0.2763 (10)	0.036 (5)*	
C14	0.72087 (12)	0.15479 (13)	0.44955 (7)	0.0220 (3)	
C15	0.84103 (15)	0.10914 (17)	0.41681 (10)	0.0351 (3)	
H15A	0.907 (2)	0.149 (2)	0.4470 (12)	0.056 (6)*	
H15B	0.846 (2)	0.008 (2)	0.4173 (12)	0.058 (6)*	
H14C	0.8516 (19)	0.147 (2)	0.3658 (12)	0.051 (6)*	
C16	0.71490 (14)	0.31393 (14)	0.58331 (7)	0.0282 (3)	
H16A	0.723453	0.384753	0.620421	0.042*	
H16B	0.791792	0.260036	0.580824	0.042*	
H16C	0.645041	0.253596	0.595442	0.042*	
C17	0.60967 (13)	0.59215 (14)	0.32293 (7)	0.0245 (3)	
C18	0.63109 (13)	0.80140 (14)	0.54760 (7)	0.0231 (3)	
H18A	0.6100 (17)	0.717 (2)	0.5740 (10)	0.037 (5)*	
H18B	0.5672 (16)	0.8694 (18)	0.5554 (9)	0.027 (4)*	
C19	0.75457 (13)	0.85366 (14)	0.57467 (7)	0.0244 (3)	
C20	0.84825 (15)	0.98717 (19)	0.66682 (9)	0.0380 (4)	
H20A	0.904 (2)	0.910 (2)	0.6807 (12)	0.053 (6)*	
H20B	0.896 (2)	1.047 (2)	0.6298 (12)	0.056 (6)*	
C21	0.80775 (18)	1.0701 (2)	0.73071 (10)	0.0410 (4)	
H21A	0.883 (2)	1.109 (2)	0.7550 (13)	0.065 (7)*	
H21B	0.762 (2)	1.014 (2)	0.7639 (12)	0.051 (6)*	
H21C	0.751 (2)	1.144 (2)	0.7167 (11)	0.049 (6)*	

### Atomic displacement parameters (Å^2^)

**Table:**

	*U*^11^	*U*^22^	*U*^33^	*U*^12^	*U*^13^	*U*^23^
S1	0.03027 (19)	0.01732 (17)	0.02199 (18)	0.00121 (12)	−0.00303 (12)	−0.00091 (11)
O1	0.0316 (5)	0.0202 (5)	0.0367 (6)	0.0000 (4)	0.0043 (4)	0.0046 (4)
O2	0.0248 (5)	0.0464 (7)	0.0389 (6)	0.0060 (4)	−0.0025 (4)	−0.0121 (5)
O3	0.0289 (5)	0.0356 (6)	0.0266 (5)	0.0020 (4)	−0.0059 (4)	−0.0092 (4)
N1	0.0257 (6)	0.0202 (5)	0.0208 (6)	−0.0021 (4)	−0.0033 (4)	−0.0004 (4)
N2	0.0473 (8)	0.0295 (6)	0.0285 (7)	−0.0007 (6)	−0.0097 (6)	0.0023 (5)
C1	0.0199 (6)	0.0200 (6)	0.0209 (6)	−0.0030 (5)	−0.0009 (5)	−0.0009 (5)
C2	0.0203 (6)	0.0180 (6)	0.0224 (6)	−0.0021 (5)	−0.0018 (5)	0.0007 (5)
C3	0.0224 (6)	0.0201 (6)	0.0220 (7)	−0.0025 (5)	−0.0032 (5)	0.0008 (5)
C4	0.0199 (6)	0.0192 (6)	0.0211 (6)	−0.0021 (5)	−0.0023 (5)	−0.0002 (5)
C5	0.0223 (6)	0.0203 (6)	0.0199 (6)	−0.0022 (5)	−0.0033 (5)	0.0012 (5)
C6	0.0283 (7)	0.0222 (6)	0.0195 (6)	−0.0010 (5)	−0.0012 (5)	−0.0010 (5)
C7	0.0281 (7)	0.0245 (7)	0.0193 (6)	−0.0024 (5)	0.0000 (5)	−0.0010 (5)
C8	0.0233 (6)	0.0230 (6)	0.0201 (6)	−0.0001 (5)	−0.0010 (5)	−0.0028 (5)
C9	0.0271 (7)	0.0233 (7)	0.0232 (7)	−0.0021 (5)	−0.0012 (5)	0.0003 (5)
C10	0.0290 (7)	0.0329 (7)	0.0209 (7)	0.0010 (6)	−0.0022 (5)	0.0007 (6)
C11	0.0337 (7)	0.0323 (7)	0.0226 (7)	0.0009 (6)	−0.0057 (6)	−0.0064 (6)
C12	0.0418 (8)	0.0230 (7)	0.0287 (8)	−0.0048 (6)	−0.0045 (6)	−0.0046 (6)
C13	0.0368 (8)	0.0240 (7)	0.0228 (7)	−0.0035 (6)	−0.0018 (6)	−0.0006 (5)
C14	0.0244 (6)	0.0202 (6)	0.0214 (6)	−0.0002 (5)	−0.0042 (5)	−0.0005 (5)
C15	0.0311 (8)	0.0294 (8)	0.0448 (10)	0.0052 (6)	0.0068 (7)	0.0029 (7)
C16	0.0393 (8)	0.0239 (7)	0.0214 (7)	−0.0016 (6)	−0.0065 (6)	0.0022 (5)
C17	0.0293 (7)	0.0211 (6)	0.0231 (7)	−0.0022 (5)	−0.0044 (5)	−0.0016 (5)
C18	0.0257 (7)	0.0207 (6)	0.0230 (7)	0.0004 (5)	0.0007 (5)	−0.0027 (5)
C19	0.0277 (7)	0.0223 (6)	0.0231 (6)	0.0038 (5)	−0.0038 (5)	−0.0008 (5)
C20	0.0329 (8)	0.0429 (9)	0.0383 (9)	0.0026 (7)	−0.0140 (7)	−0.0119 (7)
C21	0.0458 (10)	0.0446 (10)	0.0324 (9)	−0.0011 (8)	−0.0100 (7)	−0.0095 (7)

### Geometric parameters (Å, º)

**Table:**

S1—C4	1.7581 (13)	C9—H9	0.960 (19)
S1—C18	1.7918 (14)	C10—C11	1.389 (2)
O1—C14	1.2112 (16)	C10—H10	0.96 (2)
O2—C19	1.2026 (17)	C11—C12	1.381 (2)
O3—C19	1.3417 (17)	C11—H11	0.98 (2)
O3—C20	1.4578 (18)	C12—C13	1.388 (2)
N1—C4	1.3301 (17)	C12—H12	0.956 (19)
N1—C3	1.3482 (17)	C13—H13	1.003 (19)
N2—C17	1.1473 (19)	C14—C15	1.4946 (19)
C1—C2	1.4025 (18)	C15—H15A	0.98 (2)
C1—C5	1.4038 (18)	C15—H15B	0.99 (2)
C1—C6	1.4723 (18)	C15—H14C	1.02 (2)
C2—C3	1.4032 (18)	C16—H16A	0.9800
C2—C14	1.5130 (17)	C16—H16B	0.9800
C3—C16	1.5013 (18)	C16—H16C	0.9800
C4—C5	1.4032 (18)	C18—C19	1.5061 (19)
C5—C17	1.4378 (18)	C18—H18A	0.988 (19)
C6—C7	1.332 (2)	C18—H18B	0.965 (17)
C6—H6	0.968 (19)	C20—C21	1.500 (2)
C7—C8	1.4669 (18)	C20—H20A	1.00 (2)
C7—H7	0.961 (18)	C20—H20B	1.04 (2)
C8—C13	1.3958 (19)	C21—H21A	1.00 (2)
C8—C9	1.4016 (19)	C21—H21B	0.96 (2)
C9—C10	1.385 (2)	C21—H21C	0.97 (2)
			
C4—S1—C18	102.24 (6)	C12—C13—H13	120.2 (10)
C19—O3—C20	115.85 (11)	C8—C13—H13	119.0 (10)
C4—N1—C3	118.68 (11)	O1—C14—C15	122.21 (12)
C2—C1—C5	116.93 (11)	O1—C14—C2	119.95 (12)
C2—C1—C6	124.86 (12)	C15—C14—C2	117.77 (12)
C5—C1—C6	118.14 (11)	C14—C15—H15A	105.7 (13)
C1—C2—C3	119.21 (12)	C14—C15—H15B	109.9 (13)
C1—C2—C14	122.32 (11)	H15A—C15—H15B	110.5 (18)
C3—C2—C14	118.47 (11)	C14—C15—H14C	111.5 (12)
N1—C3—C2	122.74 (12)	H15A—C15—H14C	107.7 (17)
N1—C3—C16	114.90 (11)	H15B—C15—H14C	111.2 (17)
C2—C3—C16	122.34 (12)	C3—C16—H16A	109.5
N1—C4—C5	122.14 (12)	C3—C16—H16B	109.5
N1—C4—S1	120.40 (10)	H16A—C16—H16B	109.5
C5—C4—S1	117.46 (10)	C3—C16—H16C	109.5
C4—C5—C1	120.22 (12)	H16A—C16—H16C	109.5
C4—C5—C17	119.59 (12)	H16B—C16—H16C	109.5
C1—C5—C17	120.16 (12)	N2—C17—C5	178.94 (16)
C7—C6—C1	124.98 (12)	C19—C18—S1	113.86 (10)
C7—C6—H6	120.3 (11)	C19—C18—H18A	108.7 (11)
C1—C6—H6	114.6 (11)	S1—C18—H18A	107.8 (11)
C6—C7—C8	126.26 (13)	C19—C18—H18B	110.0 (10)
C6—C7—H7	118.6 (11)	S1—C18—H18B	106.7 (10)
C8—C7—H7	115.1 (11)	H18A—C18—H18B	109.7 (14)
C13—C8—C9	118.47 (12)	O2—C19—O3	124.17 (13)
C13—C8—C7	118.34 (12)	O2—C19—C18	126.38 (13)
C9—C8—C7	123.18 (12)	O3—C19—C18	109.43 (11)
C10—C9—C8	120.64 (13)	O3—C20—C21	107.39 (14)
C10—C9—H9	119.7 (11)	O3—C20—H20A	108.7 (13)
C8—C9—H9	119.7 (11)	C21—C20—H20A	112.2 (13)
C9—C10—C11	120.03 (13)	O3—C20—H20B	110.0 (12)
C9—C10—H10	118.6 (12)	C21—C20—H20B	111.1 (12)
C11—C10—H10	121.4 (12)	H20A—C20—H20B	107.4 (17)
C12—C11—C10	119.98 (13)	C20—C21—H21A	109.1 (13)
C12—C11—H11	119.8 (11)	C20—C21—H21B	110.5 (13)
C10—C11—H11	120.2 (11)	H21A—C21—H21B	109.7 (18)
C11—C12—C13	120.18 (13)	C20—C21—H21C	111.6 (12)
C11—C12—H12	120.1 (11)	H21A—C21—H21C	110.1 (18)
C13—C12—H12	119.7 (11)	H21B—C21—H21C	105.8 (18)
C12—C13—C8	120.69 (13)		
			
C5—C1—C2—C3	2.15 (18)	C5—C1—C6—C7	−140.67 (14)
C6—C1—C2—C3	−175.01 (12)	C1—C6—C7—C8	173.53 (13)
C5—C1—C2—C14	−176.70 (11)	C6—C7—C8—C13	172.91 (14)
C6—C1—C2—C14	6.15 (19)	C6—C7—C8—C9	−8.2 (2)
C4—N1—C3—C2	1.39 (19)	C13—C8—C9—C10	0.5 (2)
C4—N1—C3—C16	−179.73 (12)	C7—C8—C9—C10	−178.32 (13)
C1—C2—C3—N1	−3.20 (19)	C8—C9—C10—C11	0.0 (2)
C14—C2—C3—N1	175.69 (12)	C9—C10—C11—C12	−0.6 (2)
C1—C2—C3—C16	177.99 (12)	C10—C11—C12—C13	0.6 (2)
C14—C2—C3—C16	−3.12 (19)	C11—C12—C13—C8	0.0 (2)
C3—N1—C4—C5	1.39 (19)	C9—C8—C13—C12	−0.5 (2)
C3—N1—C4—S1	−178.47 (10)	C7—C8—C13—C12	178.38 (14)
C18—S1—C4—N1	−5.66 (12)	C1—C2—C14—O1	−114.75 (15)
C18—S1—C4—C5	174.48 (10)	C3—C2—C14—O1	66.40 (17)
N1—C4—C5—C1	−2.31 (19)	C1—C2—C14—C15	68.12 (17)
S1—C4—C5—C1	177.55 (10)	C3—C2—C14—C15	−110.73 (15)
N1—C4—C5—C17	175.55 (12)	C4—S1—C18—C19	98.96 (10)
S1—C4—C5—C17	−4.59 (17)	C20—O3—C19—O2	0.6 (2)
C2—C1—C5—C4	0.44 (18)	C20—O3—C19—C18	179.32 (12)
C6—C1—C5—C4	177.79 (12)	S1—C18—C19—O2	−26.34 (19)
C2—C1—C5—C17	−177.42 (12)	S1—C18—C19—O3	154.93 (10)
C6—C1—C5—C17	−0.06 (18)	C19—O3—C20—C21	179.23 (14)
C2—C1—C6—C7	36.5 (2)		

### Hydrogen-bond geometry (Å, º)

**Table:**

*D*—H···*A*	*D*—H	H···*A*	*D*···*A*	*D*—H···*A*
C15—H15*A*···O2^i^	0.98 (2)	2.56 (2)	3.375 (2)	139.9 (17)
C18—H18*B*···O1^ii^	0.965 (17)	2.493 (17)	3.2989 (17)	140.9 (13)

Symmetry codes: (i) −*x*+2, −*y*+1, −*z*+1; (ii) −*x*+1, −*y*+1, −*z*+1.
